# Iron Complexes of Flavonoids-Antioxidant Capacity and Beyond

**DOI:** 10.3390/ijms22020646

**Published:** 2021-01-11

**Authors:** Zdeněk Kejík, Robert Kaplánek, Michal Masařík, Petr Babula, Adam Matkowski, Petr Filipenský, Kateřina Veselá, Jakub Gburek, David Sýkora, Pavel Martásek, Milan Jakubek

**Affiliations:** 1 Department of Paediatrics and Inherited Metabolic Disorders, First Faculty of Medicine, Charles University and General University Hospital in Prague, CZ-121 08 Prague, Czech Republic; Zdenek.Kejik@lf1.cuni.cz (Z.K.); RobertKaplanek@lf1.cuni.cz (R.K.); Michal.Masarik@lf1.cuni.cz (M.M.); Katerina.vesela@lf1.cuni.cz (K.V.); David.Sykora@vscht.cz (D.S.); Pavel.Martasek@cuni.cz (P.M.); 2 BIOCEV, First Faculty of Medicine, Charles University, Prague, CZ-252 50 Vestec, Czech Republic; 3 Department of Analytical Chemistry, Faculty of Chemical Engineering, University of Chemistry and Technology, CZ-166 28 Prague, Czech Republic; 4 Department of Physiology, Faculty of Medicine, Masaryk University, Kamenice 5, 625 00 Brno, Czech Republic; Petr.Babula@med.muni.cz; 5 Department of Pathological Physiology, Faculty of Medicine, Masaryk University, Kamenice 5, 625 00 Brno, Czech Republic; 6 Department of Pharmaceutical Biology and Botany, Wroclaw Medical University, Borowska 211, 50556 Wroclaw, Poland; bbsekret@umed.wroc.pl; 7 Department of Urology, St. Anne’s University Hospital Brno, Pekařská 53, 656 91 Brno, Czech Republic; petr.filipensky@gmail.com; 8 Department of Pharmaceutical Biochemistry, Wroclaw Medical University, Borowska 211A, 50556 Wroclaw, Poland; jakub.gburek@umed.wroc.pl

**Keywords:** flavonoids, iron ions, metallocomplexes

## Abstract

Flavonoids are common plant natural products able to suppress ROS-related damage and alleviate oxidative stress. One of key mechanisms, involved in this phenomenon is chelation of transition metal ions. From a physiological perspective, iron is the most significant transition metal, because of its abundance in living organisms and ubiquitous involvement in redox processes. The chemical, pharmaceutical, and biological properties of flavonoids can be significantly affected by their interaction with transition metal ions, mainly iron. In this review, we explain the interaction of various flavonoid structures with Fe(II) and Fe(III) ions and critically discuss the influence of chelated ions on the flavonoid biochemical properties. In addition, specific biological effects of their iron metallocomplexes, such as the inhibition of iron-containing enzymes, have been included in this review.

## 1. Introduction

Flavonoids represent a group of secondary (specialized) metabolites (approx. up to 10,000) that are widely distributed in the plant kingdom. Collectively, they are a subclass of phenolics and remain the most intensively studied group of polyphenols, responsible for many health benefits attributable to high vegetable consumption and using polyphenol-rich herbs.

**Figure 1:**
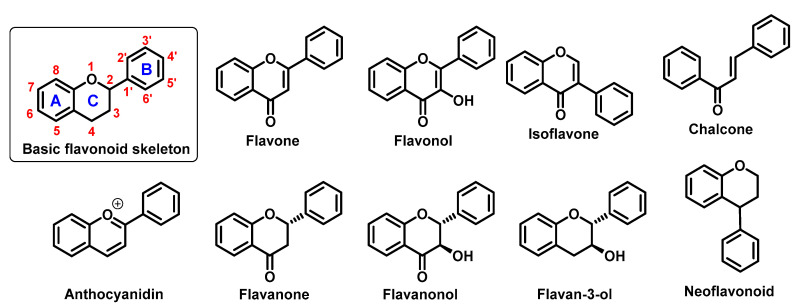
Basic flavonoid structure showing rings A, B and C and the numbering, flavonoids and chalcone chemical structures.

They perform a variety of functions, mainly ecological (giving a characteristic colour to plant organs, especially flowers and fruits), and they also participate in the regulation of plant development and growth, as well as plant–microbe and plant–animal interactions (signalling functions) [1]. Due to strong antioxidant properties, they are involved in UV protection. They are intensively discussed and studied thank their positive influence on human health. Flavonoids occur both in free state and as glycosides (*O-* or *C-*). The structure of flavonoids consists of a diphenylpropane (C6–C3–C6, benzo-γ-pyrone) skeleton [2], but biosynthetically, they originate from the general phenylpropanoid pathway complemented with malonyl-CoA to form the final core structure of flavane. Further, when the B ring is moved from position 2 of the C-ring to the carbon atom in position 3, the isoflavones are formed, whereas those in which the B ring is linked in position 4 are called neoflavonoids. However, B ring remains in position 2 of the basic flavane skeleton which can be subdivided into different subgroups depending the degree of unsaturation and oxidation of the C ring. These subgroups are: flavones, flavonols, flavanones, flavanonols, flavanols or catechins, and chalcones (you can see in Figure 1) [3].

Specific flavonoids form can be represent by anthocyanidins and anthocyanins (their glycosylated form), derivates of 2-phenylbenzopyrylium cation [4]. Unlike other flavonoids, they are intensive pigments, and are responsible for the red, blue and purple color of many plant parts. The most common anthocyanidins are cyanidin, delphinidin, peonidin, malvidin, petunidin, and pelargonidin.

It has been repeatedly postulated that these compounds can be beneficial in the prevention of numerous diseases, particularly oncological, cardiovascular, metabolic and neurodegenerative diseases [5,6,7,8,9,10,11].

Numerous works proved various anticancer effects of flavonoids. For example, quercetin can reduce can activity of a nuclear factor kappa B and expression of the P-glycoprotein and thereby angiogenesis and multidrug resistance and cell migration [12,13,14]. Similarly, Gates et al. publish, that kaempferol can significantly decrease a risk of ovarian cancer [15]. Other important benefit of flavonoid application could be the reduction of drug toxicity. Mojžíšová et al. found, that quercetin decrease daunorubicin-induced toxicity for cardiomyoblasts [16].

In this case of cardiovascular diseases, therapeutic effects of flavonoids (e.g., quercetin, or kaempferol) include antihypertensive properties and improved endothelial functions [17,18]. Hang et al. report that patients with rheumatoid arthritis after bajcalin treatment have decreased levels of apolipoproteins, triglycerides, as well as total- and low density lipoprotein cholesterol, and a lower risk of the coronary artery disease [19]. Bakker et al. found that Montmorency cherry supplement (235 mg/per day anthocyanins) enhanced the recovery of an endothelium-dependent vasodilatation after ischemia-reperfusion injury [20].

Flavonoids are also promising agents for the treatment and prevention of metabolic diseases. Vital et al. found that cyanidin and delphinidin glucosides are potent inductors of insulin secretion [9]. It was observed that the consumption of anthocyanidin-rich extracts prevents obesity in healthy subjects, and helps to reduce the body weight of obese subjects [10]. Silveira et al. report that the daily intake of red orange juice (cyanidin glucosides as major active factors) [21] leads to an increase in serum antioxidant activity and reduction in the levels of C-reactive protein as well as total- and low-density lipoprotein cholesterol [22].

Flavonoids are intensively studied for the treatment of neurodegenerative diseases. Several longitudinal studies showed that habitual consumption of tea (source of catechin, epicatechin, and epigallocatechin gallate) inversely correlate with the onset of PD [23,24]. Catechins, such as epigallocatechin gallate displayed also numerous therapeutic effects against Alzheimer’s disease [25]. Their molecular targets include amyloid beta peptides and α-synuclein, inflammation, and elevated expression of pro-apoptotic proteins. Similarly, Fan et al. publish that the daily uptake of blackcurrant anthocyanins could be beneficial for Parkinson patients [26].

The above diseases are deeply associated with oxidative stress [27,28,29,30] and flavonoids are known as strong antioxidants due to the presence of several phenolic hydroxyl groups, which can be easily oxidized [31]. Structure–activity studies have demonstrated that the antiradical/antioxidant activities are related to structural criteria, such as the presence of an ortho-hydroxyl on the B-ring, presence of one or more free hydroxyl groups, C2–C3 double bond in the C-ring, or the presence of a 3-hydroxyl group [32]. As the result, they can effectively protect cells against oxidative damage caused by excess reactive oxygen (ROS) as well as reactive nitrogen species (RNS). A balance in ROS/RNS (e.g., hydroxyl, superoxide, nitric oxide, nitrogen dioxide, or peroxyl radicals together with non-radical hydrogen peroxide and peroxynitrite) is closely connected with the redox status of the cell that is influenced also by sulphur compounds (both sulphur-rich proteins and low-molecular thiols) and metal ions with chelating properties. Although most of results have been obtained from in vitro studies, increasing evidence indicates that flavonoids may display antioxidant functions also in vivo [33,34]. Due to the fact that they can be obtained from food in significant amounts, after ingestion of flavonoid-rich food, their blood levels can reach approximately micromolar concentrations, even if most of them are not absorbed or decomposed by gastrointestinal tract inhabiting microorganisms [35]. It implies, that their antioxidant properties can have some degree of importance in the therapy and prevention oxidative-stress related diseases. These properties are, at least partially, associated with chelation of free transition metals ions, mainly iron, which may contribute to oxidative stress exacerbation. Moreover, flavonoids can directly supress ROS itself and chelated metal ions, mainly iron ones can be significantly changed upon interaction with chelated metals.

Iron is one of the essential elements of every living organism and the most widespread transition metal. Iron participates in oxygen transport, (mainly in hem form) and is necessary for correct function of many enzymes involved in electron transfer and oxidation-reduction reactions [36]. Their various functions require two different oxidation states: Fe(II) or Fe(III), that determines their affinity to a number of biological ligands, such as amino acids, thiols, phenols and porphyrins. Most of the body iron is sequestered in the hem moiety of erythrocyte haemoglobin and muscle myoglobin (73%). Other 15% represent so called iron-labile-pool that is requested for many important ongoing biochemical reactions. The remaining 12% is stored in cellular ferritin.

A number of recent works showed that flavonoids (e.g., quercetin, baicalein, and baicalin) can form high-affinity complexes with transition metal ions, such as iron and copper [37,38,39,40]. This phenomenon is considered as the key mechanism of their biological activity (e.g., radical scavenging) [39,41,42]. Furthermore, metallocomplexes formed by iron chelation may display their own unique biological activities. Iron complexation affects their biochemical properties such as lipophilicity, membrane transport, or interactions with biomolecules [43,44,45,46,47,48,49,50,51,52,53,54,55,56].

## 2. Chelating Properties of Flavonoids

**Figure 2:**
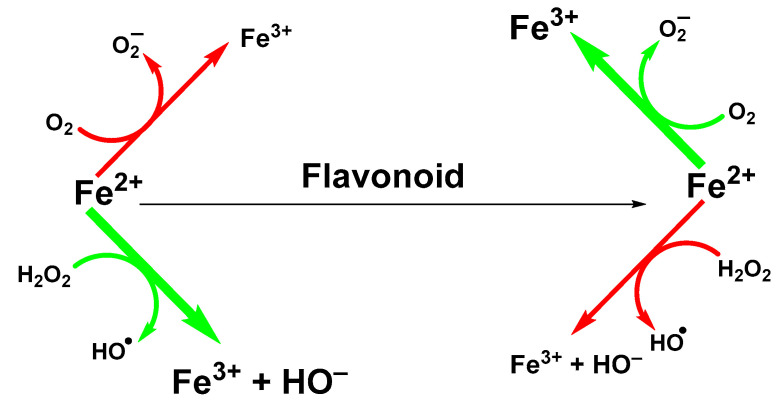
Suppression of Fenton reaction by flavonoids.

Ability of flavonoids to neutralize reactive radicals is usually linked to their antioxidant properties. However, other mechanisms can be involved too. One of them is the ability to sequestrate metal ions, such as iron and copper ions that participate in the generation of hydroxyl radicals in Fenton (Fe(II); Figure 2) or Fenton-like (Cu(II)) reactions [32].

**Figure 3:**
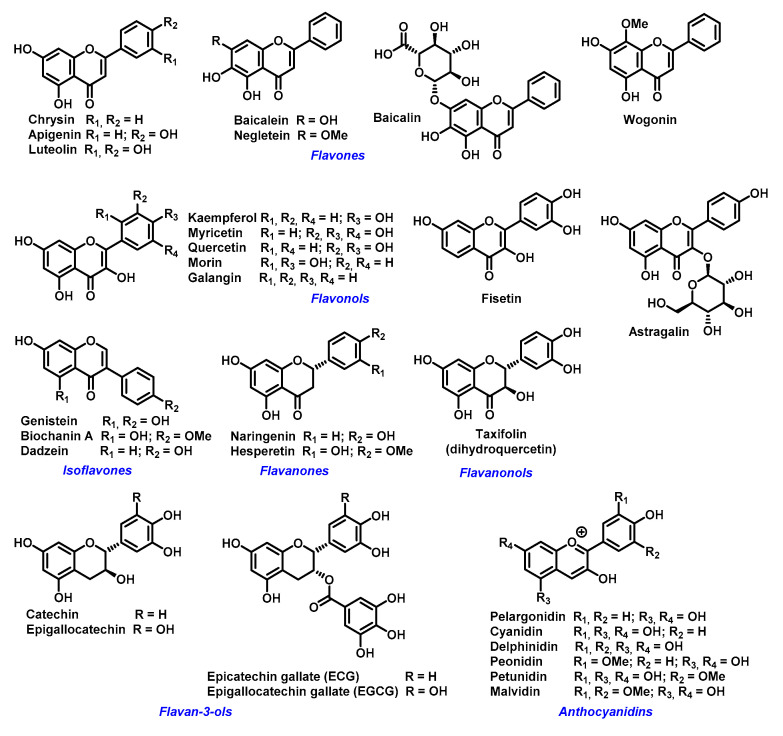
Examples of biologically active flavonoids.

The ability of flavonoids to strongly chelate these metal ions contributes to their antioxidant properties. The chelation takes place via hydroxyl groups or their carbonyl moiety, if present [57]. Next possibility how flavonoids exert antioxidant effects is to react with -SH groups with subsequent modification of redox environment inside the cell [58]. Quercetin, a wide-spread flavonol, is one of the best investigated flavonoids. Its ability to chelate a wide range of metal ions, such as Al(III), Co(II), Cr(III), Cu(II), Fe(II), Fe(III), Mo(VI), Pb(II), Tb(III), and Zn(II)) has been shown in several studies [59,60,61]. Similar data have been obtained for rutin (quercetin-3-*O*-beta-rutinoside) or catechin (flavan-3-ol) towards Cu(II), Fe(II), and Zn(II) ions [32]. However, most come from studies of flavonoids-iron ions complexes. Examples of flavonoids with high affinity for iron ions that have been used for therapeutic applications are shown in Figure 3.

### Influence of Chelated Iron Ions on the Antioxidative Effect of Flavonoids

Typically, the antioxidant activity of flavonoids is based on reduction (scavenging) of oxygen radicals. Often, their protective effect towards biomolecules, such as inhibition of lipid peroxidation can correlate with their antiradical activity as based on either reducing the initial ROS or breaking the chain reaction of propagating peroxyl radicals among polyunsaturated fatty acid molecules and other intermediates [62]. Moreover, flavonoids can suppress the production of ROS due to the inhibition of enzymes which control redox balance and inflammatory processes such as: cyclooxygenases, lipoxygenases, xanthine oxidase, NADH oxidase, SOD, catalase, and myeloperoxidase [63,64,65,66,67,68].

Flavonoids, with their multiple hydroxyl groups and the carbonyl group on ring C (flavones), have several available sites for metal complexation. Miličević et al. noticed a strong correlation between polyphenols’ antioxidative activity and their affinity for Fe(II) ions (represented by binding constants), suggesting that the suppression of Fenton reaction is most probably caused by chelation of the ion [39]. Porfírio et al. found that Fe(II) complex of morin, quercetin and fisetin (most probably 1:1) displayed higher antioxidant capacity for Ce(IV) (about 15%, 32% and 28%, respectively) than flavonoids alone [42]. Kostyuk et al. found that metal complexes of rutin, taxifolin, epicatechin, luteolin with Fe (II), Fe(III), and Cu(II) ion have significantly higher activity than free flavonoids [41]. Also, all complexed flavonoids were oxidized significantly less than free flavonoids. However, the activity of EGCG complex with Fe(II) ion was reduced to a quarter, while activity of Cu(II) complex was more than double [69]. Similarly, Dowling et al. report [70] that isoflavone-copper chelates (biochanin A and genistein, 2:1) displayed higher antioxidative activity than the free compounds, whereas their complexes with Fe(III) had higher pro-oxidant activity.

On the other hand, the chelation ability can be strongly influenced by their reducing properties. For example, Mira et al. observed, that only myricetin and quercetin (flavonols with significant reducing activity) had strong affinity for Fe(III) ions [71]. Similarly, Loizo et al. observed at pH 7.2, a partial reduction of chelated Fe(III) by flavonoid compounds [72]. It implies that chelation is more effective, when the metal ion is in its bivalent form, and most their complexes with transition metal ions in the living system contain Fe(II) ions.

However, metal chelation depends on the pH, solvent (polarity and ionic composition),) and stoichiometry (flavonoid to iron ratio). Rutin and negletein were active inhibitors of the Fenton reaction at very low ratios, while being pro-oxidant or ineffective in the proximity of a 1:1 ratio [73]. Nevertheless, pure ferric salt or ferric-ADP or ferric citrate do not intensify the Fenton reaction in the presence of flavonoids [74,75].

A couple of structural features influence the complexation ability of flavonoids, but the number and position of hydroxyl groups are crucial [76]. In general, flavonoids with the 6,7-dihydroxy pattern exhibit strong complexation ability in neutral and acidic pH. Flavonols with 3-hydroxyl group, the 4-ketogroup and the 2,3-double bond with the catecholic B ring are strong chelators in the neutral and slightly acidic pH and flavonoids with 5-hydroxyl-4-keto chelation site are weaker chelators even in the neutral pH. Fe(II) may interact not only on the deprotonation sites but also on the other positions that originate from resonance effects. Thus, several possible complexes are possible (1:1, 2:1, 1:3 and 2:3, Fe (II): flavonoid) [77]. For example, quercetin has three possible binding sites for the chelation of Fe(II) ions [78]. MS studies (electrospray ionization mass spectrometry) implied that the preferred flavonoid complexation site is hydroxyl at carbons 3 or 5 and the adjacent 4-carbonyl group [79].

Electron density is an important factor in the interaction of flavonoids with metal ions. In an aprotic solvent its influence can be small. For example, in the DMSO (aprotic polar solvent) both neutral and monoanionic quercetin with Fe(II) ions forms 1:1 complexes [80]. Conversely, a protic solvent such as water, can interact with phenyl and carbonyl group of a flavonoid and depending pH control their dissociation and thereby their interaction with metal ions. For example, biochanin A and genistein prefer 1:2 stoichiometry of their complexes with Fe(III) ions in the acidic and neutral pH, whereas in the basic pH another phenyl group can be deprotonated and 1:1 complex is formed [70]. Similarly, naringenin form complex with Fe(II) ion with 2:1 and 1:1 stoichiometry at the pH 7.4 and 9, respectively [81]. Flavonoids with higher number of hydroxyl groups such as taxifolin form complex with 1:2 and 2:1 stoichiometry at acidic pH (5), whereas 2:1 complex is observed for the neutral and basic pH. An influence of surrounding ions, e.g., in the used buffer, can also be important. It is well known that phosphate anions form strong complex with iron ions and thereby supress their interaction with other molecules. In a living system, such as human body phosphate concentration can be relatively high. In plasma of adults, phosphate concentration is around 0.3 mmol/L [82]. In erythrocytes, the phosphate level can be in millimolar values [83]. Value of binding constants obtained from titration in the presence of phosphate buffer (tens of millimoles, see Table 1) implies their chelation effect in the living system is a highly probable phenomenon. Quercetin application led to the dissociation of equimolar Fe(II) ferrozine complex (K = 3.65 × 10^15^ M^−3^) [84] and formation of Fe(II)-qeurcetin complex in the 20 mM phosphate buffer [37].

Another points, which prove iron chelation by flavonoids in vivo can be given by anthocyanins [85]. A complexation anthocyanins (e.g., cyanidin, delphinidin, peonidin, malvidin, or their glucosides) of with transition metal ions such as iron cause bathochromic shift and increase intensity of their color and their stability [86]. Hover, co in the neutral pH this shift can be strongly reduced, but complexation can be observed [87]. Nevertheless, their natural environment displayed rather acidic pH. We can expect that a significant part of these compounds is in the form of metallocomplexes. For example, Kunsagi, et al. calculated free enthalpy and found for the formation of malvidin–Fe(II) complex (1:1, phosphate puffer, pH = 3.2) 49.12 kJ/mol [88]. The value of binding energy is enough to support this hypothesis. Nevertheless, it was shown, in the plants, that anthocyanins form complicated supramolecular complexes with other flavonoids and metal ions [89]. It is implied that some known biological effect of flavonoids can be also explained by formation of supramolecular complexes via a coordination of metal ion/ions.

In the biological systems or models such as a synthetic membrane, other flavonoid properties such as hydrophobicity, membrane permeability and thereby biological effectivity can be at least equally important as their Fe(II) affinity [90,91,92]. On the other hand, metal chelation can influence other flavonoid properties important for their biological activity such as pKa and hydrophobicity. For example, in the case of catechin derivatives, their complexes with Fe(II) and Fe(III) ions have lower pKa than the non-complexed compounds, except Fe(III) complex with EGC [69].

Using molecular models or experimental studies, we suggested that the flavonoids lipophilicity may considerably increase after iron chelation. Kim et al. show that lipophilicity of simple complex is approximately half of that of free quercetin. The lipophilicity of 1:2 and 2:3 complex (metal:ligand) is two and three times higher, respectively [53]. On the other hand, some works implied that quercetin in the membrane system can improve its availability for Fe(II) with subsequent complex formation [54].

In hydrophobic solvent (octanol), stoichiometry (e.g., with a highly hydroxylated flavanonol such as taxifolin) can change to more hydrophobic complex (from 1:1 to 1:3) [55]. This process enables incorporation of hydrophilic metal ions to hydrophobic surroundings and support flavonoids interaction with hydrophobic system such as membranes and liposomes. For example, Tarahovsky at al. observed that Fe(II) flavonoid complexes (quercetin, taxifolin, catechin, and morin) support liposomes aggregation [93].

Generally, we can say that flavonoid’s ability for ROS suppression, if we do not consider their effect on enzyme activity, is based mainly on their chelation of Fe(II) ions, optionally preceded by reduction of Fe(III) ions [39,71,72]. This effect is influenced by other factors such as pH and polarity of the reaction milieu [80,81]. On the other hand, the chelation of Fe(II) ions can significantly influences flavonoid properties, such as anti ROS effectivity, hydrophobicity, membrane permeability, and thereby their physiological activity [41,43,53].

## 3. Flavonoid Metallocomplexes in the Living Systems and Biological Effect of Their Interactions with Iron Ions

Iron-flavonoid complexes can interact with various biomolecules, hence the importance of research aimed at their potential biological and medicinal applications. For example, Yang et al. found that flavonoid affinity for HSA is significantly improved in the presence metal ions (e.g., Fe(II) and Co(II)) [52]. Also, part of the hemoglobin complexation with an isoflavonoid such as genistein is its interaction with hem iron [51].

**Figure 4:**
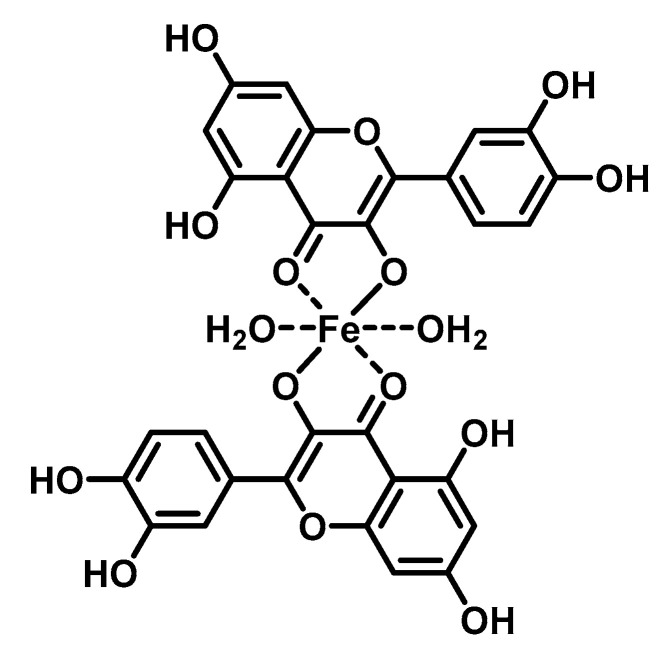
Structure of tested iron-quercetin complex.

The DNA-flavonoid metallocomplexes displayed significantly higher affinity than free flavonoids. For some of them such as quercetin-zinc complex (2:1, ligand:metal), intercalation into DNA was observed, resulting in significant improvement of cytotoxicity to cancer cell lines (HepG2, SMMC-7721, and A549) [94]. Raza et al. showed that an enhanced antibacterial effect (e.g., *Staphylococcus aureus*) of quercetin-iron complex (2:1, ligand:metal; Figure 4) could be associated with DNA intercalation and hydrolysis [49].

On the other hand, that metal complexes of flavonoids (rutin, dihydroquercetin, epigallocatechin gallate and epigallocatechin) displayed strong in vitro protective effect against chrysotile asbestos-induced hemolysis [56]. For example, combination of FeSO_4_ with rutin or dihydroquercetin unharmed red cells from oxidative stress an order of magnitude higher than free flavonoids. According to the authors, this effect could be related to the enhanced membrane uptake of these metallocomplexes.

Baccan et al. reported that quercetin iron complexes can cross cell membrane of iron-overloaded HeLa cells (10 μM (NH_4_)_2_Fe(SO_4_)_2_ and 100 μM ascorbate) and transport the ions to transferrin [43]. A possible explanation of this phenomenon could be a formation of hydrophobic Fe(II) metallocomplex with more quercetin ligands. On the other hand, Horniblow et al. observed that formation of quercetin-iron complex hindered iron uptake into the RKO cells (human colon carcinoma cell line) [38]. However, preincubation with quercetin (12 h before FeSO_4_ application) resulted in ion higher labile intracellular Fe(II) level than without quercetin. This effect was coupled with significant reduction of ferritin expression both with quercetin and rutin and increased TfR1 level (transferrin receptor protein 1; required for iron import from transferrin into cells). This observation phenomena could be explained by higher cellular transport and lover storage capacity. Nevertheless, simultaneous application of FeSO_4_ with small amount of the quercetin (2 μmol/L) lead to higher concentration of Fe(II) ions, against alone FeSO_4_ but when rutin was used, the effect was observed dose-dependently at 2, 20 and 200 µmol/L. A plausible mechanism was suggested by Vlachodimitropoulou et al [91]. who found that GLUT1 (glucose transporter) can transport quercetin–Fe(II) (in this case small quercetin concentration; 1 μmol/L and less) from cytosol to extracellular medium. At the same time, quercetin, but not rutin, is also a GLUT1 inhibitor by binding to its exofacial site that would explain the irregular dose-response [95].

**Figure 5:**
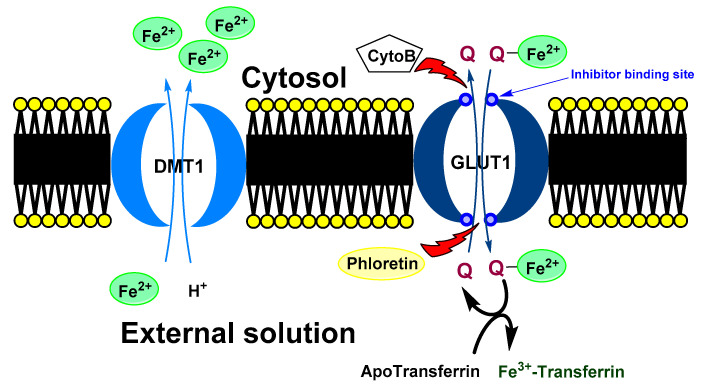
Possible mechanism of quercetin effect on intracellular iron homeostasis. Ferrous ions are usually transport to a cell by DMT1. In the cytosol, quercetin can chelate iron ions. Formed complex is transported out the cell via GLUT1. In the opposite direction, free quercetin is transported to a cell. In the extracellular space, chelated iron ion is moved from quercetin to the transferrin (blood transport protein for the ferric ions). In the shortly, quercetin can decrease intercellular concentration of iron ions. DMT1 (divalent metal transporter 1), GLUT1 (glucose transporter), Cyto B (cytochalasin B) and Phloretin (Inhibitors of GLUT1), Q (quercetin).

A proposed model of quercetin effect on the intracellular iron homeostasis is shown in Figure 5.

It implies that their biological effect can be significantly different from both components (a free flavonoid and free iron ions). For example, in the study by Farid at al [46], the application of iron-quercetin complex lead to significant increase of catalase activity and reduction of protein carbonylation and level of thiobarbituric acid reactive substances in the adipose tissue of diabetic rats. The effect of only free Fe(II) ions in the form of iron sulphate or alone quercetin was opposite. It is plausible that some physiological effects attributed to quercetin are actually caused by its complexes with metal ions, such as Fe(II). For example, quercetin-iron complex facilitated the formation of NO from nitrite both in vitro and in vivo. Although, the quercetin itself can react with nitrite to form NO in vitro [96,97], this process can be limited in vivo. Raza et al. found that application of nitrite along with quercetin and FeSO_4_ increased formation of NO-hem complexes in rat erythrocyte haemoglobin, whereas the effect of FeSO_4_ and quercetin alone was significantly lower or negligible [48].

The evidence summarized above suggests that investigating biological effect of flavonoids should consider not only the original and intact molecular structure. Besides metabolic transformation (e.g., demethylation, deglycosylation and sulfonation of phenyl groups) [98,99,100,101,102], formation of flavonoid-metal complexes, in particular with iron and other transition biometals significantly modifies their properties. Full understanding of these interactions would certainly lead to their adequate application in prevention and treatment of many health disorders.

### 3.1. Influence of Flavonoids on Iron Dependent Enzymes

Other potentially therapeutic effect of polyphenols Fe(II) includes inhibition of Fe(II) dependent enzymes such as Jumonji histone demethylase, lypoxygenases and prolyl hydroxylase (inhibition activity of flavonoids are shown in Table 2).

The Jumonji histone demethylase catalyses the demethylation of trimethyl lysine 9 of histone H3 [103]. Its targeting could be a feasible way for the treatment of some oncological diseases such as androgen-dependent prostate cancer [104]. According to Sakurai et al. some compounds with polyphenolmotifs (e.g., myricetine, baicalein, epigallocatechin, epigallocatechin gallate, dopamine hydrochloride and isoproterenol hydrochloride) inhibited Jumonji histone demethylase and prolyl hydroxylases [50]. This effect can be explained by simple complexation of Fe(II) ions. However, in some cases, an increase of Fe(II) level eight times led to only slight increase of enzyme activity, for example for myricetin and epigallocatechin it was only 60 and 10 %, respectively. More importantly, for some compounds such as L-methylDopa, 6-hydroxy-DL-Dopa, dopamine hydrochloride, isoproterenol hydrochloride the enzyme activity decreased with higher Fe(II) concentrations.

Lipoxygenases, which contain Fe(III) ions in their active site, catalyse conversion of polyunsaturated fatty acids (e.g., linoleic acid and arachidonic acid) to hydroxylated fatty acids [105]. They are classified depending on the position of dioxygenation. Their dysregulation is associated with inflammation-based pathogenic processes, e.g., in cancer or atherosclerosis.

Many flavonoids are potent lypooxygenase inhibitors. For example, soy lypoxoygenase-1 and human lypoxoygenase-5 (from polymorph nuclear lymphocyte) were inhibited by genistein (IC_50_ = 136 and 157 nmol/L) and daidzein (IC_50_ = 107 and 125 nmol/L, respectively). The inhibition mechanism, confirmed using electron paramagnetic resonance is based on reduction of active Fe(III) ion to Fe(II) and stopping its re-oxidation [63]. The activity of 7-O-glycosides of both isoflavones was identical, suggesting a crucial role of 4′-hydroxyl group in this kind of activity, but not 7-hydroxyl which is blocked by the sugar moiety. According to Mascayano at al., the mechanism of baicalein and quercetin lipoxygenase inhibition (12 and 15) relies on chelation of Fe(II) ions in the enzyme active site [47]. Despite lack of hydroxyl group at 4′ position in baicalein, the steered molecular dynamics simulation implies, that baicalein could interact with enzyme Fe(II) ions of lipoxygenase-15 via the hydroxyl group in C6 position. In quercetin, the simulation implies that quercetin binds the Fe(II) ion of lipoxygenase-12 via 3 hydroxyl group, whereas 4′ hydroxyl group support inhibition by interaction with Gln 406. The different mechanisms could explain strong difference in IC_50_ value of quercetin (0.25 and 2.6 μmol/L) and baicalein (9.1 and 0.86 μmol/L) between lipoxogenase-15 (human reticulocyte) and lipoxygenase-12 (human platelet), respectively [106]. Another important Fe(II) dependent enzyme is PHD2 (HIF-prolyl hydroxylase). PHD2 causes ubiquitination and subsequent degradation of HIF-1α (Hypoxia-inducible factor 1-alpha; an important regulator of gene expression). HIF-1α overactivity plays an important role in carcinogenesis and is generally associated with poor prognosis. It was observed that baicalein or quercetin application led to inhibition of PHD2 via chelation of Fe(II) ion in the active enzyme site [44,45]. However, an abundance of Fe(II) ions reversed inhibition of both flavonoids. Similar effect was observed for galangin, but not for chrysin and wogonin, suggesting an importance of 3-hydroxy group in the mechanism of enzyme inhibition. Inhibited degradation of HIF-1α resulting from flavonoid activity could also lead to an increased risk of carcinogenesis. On the other hand, flavonoids impair MAPK-dependent phosphorylation of HIF-1α [107,108]. This effect leads to release HIF-1α from the nucleus and loss its transcriptional activity against reduced degradation. However, more evidence is necessary to support such indirect effects that may have an enormous impact on the role of flavonoids on the whole organism level.

In conclusion, binding of Fe(II) ion in the enzyme active site by hydroxyl group of flavonoids is a rather negligible part of their biological activities even if an increase of Fe(II) usually restores enzymatic activity [44,45,50]. Yet, flavonoids significantly influence intracellular Fe(II) and the iron pool [38,43,91]. Therefore, the predictability of flavonoid influence on the activity of Fe(II/III) dependent enzymes in the living systems is limited.

### 3.2. Influence of Flavonoids on the Heme Enzymes

Another important enzyme class, which activity can be controlled by flavonoids are heme enzymes [109], such as catalase, heme peroxidases and cytochromes P450). The essential part of their active site is protoporphyrin IX or their derivatives containing coordinated Fe(II) or Fe (III) ion [110].

Aromatase (CYP450) is a key enzyme for biosynthesis of estrogen hormones [111]. It converts aliphatic androgens (testosterone and androstenedione) to the aromatic estrogens (estradiol and estrone, respectively). These hormones bind to the nuclear estrogen receptors leading to stimulation of cell proliferation. In some cancer types, the inhibition of aromatase is an intensively studied treatment method [112].

**Figure 6:**
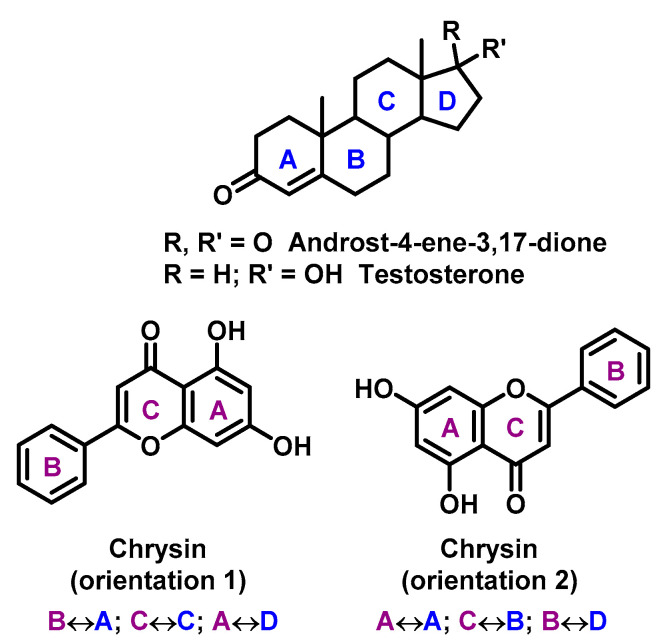
Chrysin mimic steroid core (rings in steroids are marked in blue, rings in chrisin in violet, with mutual rings overlapping).

Flavonoids are proved to be competitive inhibitors of aromatase enzyme, with IC_50_ values ranging from sub-micromolar to high-micromolar levels (inhibition activity of flavonoids are shown in Table 2) [113]. The exact molecular mechanism of the inhibition is not known. Based on structural analysis, one can expect that the A and C rings of flavonoids, such as in the chrysin structure, mimic C and D rings of the steroid core (Figure 6) [114].

Some results imply that an important part of the inhibition mechanism is the interaction of heme iron with the carbonyl oxygen at C4 position [115]. Kellis et al. reported that 7,8-benzoflavone, chrysin, apigenin, flavone, flavanone, and quercetin displayed significant activity against aromatase in order of decreasing potency [113], whereas 5,6 benzoflavane was not of any effect. Concordantly, the spectral changes (red shift in Soret band) indicated the flavonoid binding to the enzyme active site. Similarly spectral response (but significantly slower) was observed for cyclohexanone interaction with the heme moiety of cytochrome P-450 [116]. It is speculated that α-naphtolflavone structure represents an important structural motif for the aromatase inhibition. Kao et al [85] suggested that 5-hydroxyl group reduces the flavonoid binding affinity to the enzyme, probably by forming a hydrogen bond with the 4-keto group. Concordantly, the reduction of the hydroxyl group at that position caused a loss of inhibitory activity [117]. It was suggested that a suitable flavonoid compound could be a perspective inhibitor of aromatase activity in vivo. Although it appears that their administration may actually increase the enzyme activity in the living system, Sanderson et. al. observed that quercetin and genistein at concentration of 10 μmol/L induced aromatase activity in human H295R cells, 4- and 2.5-fold, respectively [118]. This phenomenon was associated with increased intracellular cAMP concentrations, subsequently inducing pII promoter-specific aromatase transcripts expression [119].

Catalase is an important part of antioxidative barrier that protects cells from the oxidative damage by ROS. The enzyme contains four identical subunits, each one associated with one molecule of ferric protoporphyrin IX.

The influence of flavonoid structure on the catalase was studied in detail by Krich et al. [67]. They found that myricetin, ECG, EGCG, sodium azide, kaempferol, catechol, gallic acid, quercetin, rutin, astragalin, galangin, apigenin, luteolin, pyrogallol, and catechin displayed significant inhibitory activity against the enzyme, in order of decreasing potency. IC_50_ values for myricetin and catechin were 0.014 and 345 μmol/L, respectively. Notably, EGCG interaction with the enzyme was associated with decrease of catalase absorbance at 405 and 630 nm with concomitant absorbance increase at 435, 530, and 575 nm, the changes characteristic for heme-flavonoid interaction. Moreover, the additional bands typical for oxidized flavonoids were also detected. On the other hand, the ferric heme of catalase is located approximately 20 A° below the protein surface, thus it is accessible only by small molecules such as hydrogen peroxide [120]. This prompts an alternative hypothesis based on conformational changes within the enzyme structure that occur upon flavonoid binding, most probably affecting the proper geometry of substrate channel that is necessary for the interaction of H_2_O_2_ with the heme centre.

It was found, that plasma concentration of flavonoids ranges between 0.03 and 9.8 μmol/L after ingestion of flavonoid-rich food [35]. It implies that the most potent catalase inhibitors (e.g., myricetin) could possibly suppress catalase activity in vivo after oral administration. However, some authors found that, flavonoids administration can stimulate catalase expression and/or activity, most probably by activation of redox-sensitive Nrf2 signalling pathway in cells [121,122]. On the other hand, some researchers observed an increase of intracellular ROS level in cells after application of flavonoid catalase inhibitors [123,124].

Myeloperoxidase is one of the most abundant protein in neutrophils, monocytes, and macrophages [125,126]. Its primary physiological function is oxidation of chloride ion to hypochlorous acid (strong oxidant) in the presence of hydrogen peroxide. Besides it can oxidize numerous low molecular substrates such as phenolic compounds. It was observed that some flavonoids are effective inhibitors of myeloperoxidase. For example, Loke et al. reported that quercetin, at the concentrations found under physiological conditions (1 μmol/L), protects low-density lipoprotein from neutrophil-mediated modification by liberated hypochlorous acid due to myeloperoxidase inhibition with IC_50_ = 1 μL [68]. According to Shiba et al. [127] the presence of the 3-, 4′-, and 5-OHs, and C_2_-C_3_ double bond are required for the inhibitory effect. The validated QSAR model indicated that the B-ring hydroxyl group(s) of flavonoids may be important for their affinity to the heme pocket containing the active site of enzyme, and that hydroxyl group of B-ring is oriented towards the heme central iron ion. Other experiments showed that, glucuronidation at the 3-position did not significantly affect quercetin inhibition [68]. However, sulfation at the 3′-position significantly reduced the inhibitory effect, and its methylation slightly decrease the enzyme activity. On the other hand, flavonoids can decrease the level of hypochlorous acid not only by inhibition of the enzyme but also by directly reacting with it to form chlorinated flavonoids (e.g., 6-mono and 6,8-dichlorinated quercetin, chlorinated genistein—in ortho position of hydroxy group) [128,129].

It was observed that macrophages treated by lipopolysaccharide (1 μg/mL) displayed significantly higher accumulation of flavonoid compounds, such as quercetin-3-glucuronide against no stimulated ones [130]. Therefore, one can expect that the uptake of these conjugates and subsequently their intracellular action is one of the mechanisms that protects inflammatory cells from the oxidative damage.

Due to their potential therapeutic importance, flavonoids have been tested in the clinical studies. Ibero-Baraibar et al. [102] showed a significant reduction of lipoprotein oxidation after flavonoid-reach extract (green tea) administration to obese volunteers, most probably caused by myeloperoxidase inhibition [131,132]. In the study of Lowe [104], green tea extract also led to a significant increase in blood antioxidant capacity [133]. Nevertheless, in this leukocyte stimulation model (analogue of bacterial peptide, fMet-Leu-Phe; whole blood), the amount of released myeloperoxidase and lactoferrin was also increased (23 and 41%, respectively).

In conclusion it seems that flavonoids are potent inhibitors of heme enzymes. However, their inhibitory effects may not always be related to direct binding with heme iron ion, as it is in the case of catalase [120]. Because of the complexity of their biological interactions the effects can be associated with other mechanisms, such as induction target enzymes by flavonoids, or influence of chlorination on their inhibition activities). The determined values of IC_50_ fell within the range of their physiological concentrations, which suggests that they can inhibit these enzymes in the living systems [35]. However, their therapeutic applications are not possible without further biological and clinical studies.

## 4. Future Direction

In the future, one can expect an increase in flavonoid-based applications in treatment and prevention of a number of serious disorders, mainly associated with oxidative stress. However, the biological interactions of chelated transition metal ions, especially iron ones, need to be more closely determined. Particularly, the risk of their potential toxicity must be taken under consideration. For example, Yen et al [134]. reported that some flavonoids, such as naringenin and hesperetin, can exert cytotoxic effects on human lymphocytes at higher levels. The authors observed an enhanced oxidation of deoxyribose by Fe(III) ions in the presence of H_2_O_2._ The generation of hydrogen peroxide and superoxide anion, as well as TBARs levels (proof of lipids peroxidation), increased in the dose-dependent manner. Accordingly, Macakova et al. found that naringin, hesperetin, and rutin have antioxidant properties at low concentrations but rather pro-oxidant at higher levels [73]. Moreover, significant flavonoids’ influence on the cell viability and DNA damage was observed for their higher concentrations [49].

On the other hand, a polyphenol chelator with reduction ability can in vitro damage DNA without during interaction with DNA. For example, Zubair et al. report that the reduction of Cu(II)to Cu(I) by genistein in breast cancer lines (MDA-MB-231 and MDA-MB-468) led to ROS generation and subsequently DNA destruction an apoptosis [135].

This phenomenon is not without significance even for a possible medicinal application of flavonoids. Some flavonoid lipoxygenase inhibitors are tested for their treatment of osteoporosis. For example, baicalein subcutaneously applications improved the cortical bone parameters in ovariectomized rats [136]. Nevertheless, iron deficiency and excess can be high risk factors for the osteoporosis, both of them can lead to bone loss [137,138]. Excess of iron stimulate degradation of bone matrix (type I collagen) and triggers severe inflammatory reactions [139]. Nevertheless, in the collagen synthesis some enzymes such as prolyl-4-hydroxylase [140] and lysyl-hydroxylase [141] need Fe(II) ions as cofactor. Deeply understanding these phenomena could help to determine limits the applicability of tested flavonoids and predict and excluded the possible side effects.

On other hand, some high impact studies strongly imply, the metallocomplexes of flavonoids have great potential as novel drug, or food supplements [46,48,49,56]. Nevertheless, for their wider applications may be necessary to optimize their pharmacological properties. One of most used methods for these purposes are formulations of bioactive compounds by suitable nano/microparticles, or molecular transport system such cyclodextrins [142,143]. From what we know, these agents have not yet been tested for flavonoid metallocomplexes. However, the result of their applications for their flavonoids formulation indicated the high potential of this approach for higher solubility (γ-CD) [144], control realising (graphene nanopartices) [145], oral application, and efficiency improvement (phospholipids) [146].

## 5. Conclusions

Significantly, part of flavonoids biological effects is based on the complexation of transition metal ions, mainly iron ones. Cited works strongly imply that their chelation ability is deeply associated with potentially beneficial preventive and therapeutic effects such as ROS neutralizing. On the other hand, chelated iron ions can significantly influence chemical, pharmaceutical and biological properties of flavonoids. Therefore, the biological effects of their iron metallocomplexes have been also presented and discussed in this review.

## Figures and Tables

**Table 1:** Binding constants of flavonoid complexes with iron ions in the phosphate buffer.

Flavonoid	Method	System	K	St.	REF.
**Fe(II)**					
Quercetin	microcalorimetery	0.1 M, pH = 7.2	3.6 × 10^6^/M	1:1	[38]
	UV-Vis	0.02 M, pH = 7.2	2 × 10^6^/M	1:1	[37]
			5 × 10^10^/M^2^	1:2	
Baicalein	UV-Vis	0.02 M, pH = 7.2	9 × 10^11^/M^2^	1:2	[40]
Rutin	microcalorimetery	0.1 M, pH = 7	3.2 × 10^8^/M	1:1	[38]
	UV-Vis	0.02 M, pH = 7.2	4 × 10^11^/M^2^	1:2	[37]
3-HF			2 × 10^11^/M^2^	1:2	
3″,4″-DHF			3 × 10^10^/M^2^	1:2	
Chrysin			8 × 10^10^/M^2^	1:2	
**Fe(III)**					
Quercetin	microcalorimetery	0.1 M, pH = 7	8.3 × 10^5^/M^2^	1:2	[38]
Rutin			2.2 × 10^4^/M^2^	1:1	

3″,4″-DHF (3″,4″-Dihydroxyflavone), 3-HF (3-Hydroxyflavone), K (Binding constant,), St (Stoichiometry; metal:flavonoid).

**Table 2:** Flavonoid inhibition activity on the iron depended and heme enzymes.

Enzymes	Flavonoids	IC_50_ (μmol/L)	Ref.
**Iron Dependent Enzymes**			
Jumonji histone demethylase	baicalein	4.0	[50]
	epigallocatechin	5.0	
	epigallocatechin gallate	3.2	
	myricetin	3.6	
soy lypoxoygenase-1	genistein	0.136	[63]
	daidzein	0.107	
human lypoxoygenase-5	genistein	0.157	
	daidzein	0.125	
lipoxogenase-15 (human reticulocyte)	baicalin	9.1	[106]
	quercetin	0.25	
lipoxygenase-12 (human platelet)	baicalin	0.86 μmol/L	
	quercetin	0.26	
**Heme Enzymes**			
Aromatase (CYP450)	7,8 benzoflavone	0.07 ^1^/0.06 ^2^	[113]
	chrysin	0.5/0.4	
	apigenin	1.2/1	
	flavone	8/5	
	flavanone	8/5	
	quercetin	12/10	
	flavone	10 ^1^	[117]
	7-hydroxyflavone	0.5	
	7,4′-dihydroxyflavone	0.2	
	flavanone	8	
Catalase	Galangin	46	[67]
	Kaempferol	1.8	
	Astragalin	45	
	Quercetin	33	
	Rutin	36	
	Myricetin	0.014	
	Catechin	345	
	Epigallocatechin	16.6	
	Epicatechin gallate	0.029	
	Epigallocatechin gallate	0.33	
	Apigenin	49	
	Luteolin	56	

^1^ Androstedione (0.04 μmol/L) as substrate; ^2^ Testosterone (0.08 μmol/L) as substrate.

## Data Availability

Not applicable.

## Abbreviations

3-HF	3-Hydroxyflavone
4″-DHF	3″,4″-Dihydroxyflavone
A549	Human non-small cell lung carcinoma cell line
ATP	Adenosine tri-phosphate
cAMP	Cyclic adenosine monophosphate
DMSO	Dimethylsulfoxide
DMT1	Divalent metal transporter 1
EGCG	Epigallocatechin gallate
ECG	Epicatechin gallate
GLUT1	Glucose transporter 1
HeLa	Human cervical cancer cell line
H295R	Human adrenocortical cell line
HepG2	Hepatoma G2 cell line
HIF-1α	Hypoxia-inducible factor 1-alpha
K	Binding constant
MDA-MB-231	Human breast cancer cell line
MDA-MB-468	Human breast cancer cell line
MAPK	Mitogen-activated protein kinase
Nrf2	Nuclear factor erythroid 2-related factor 2
PHD2	HIF-prolyl hydroxylase
RKO	Human colon carcinoma cell line
RNS	Reactive nitrogen species
ROS	Reactive oxygen species
SMMC-7721	Human hepatocellular cell line
St	Stoichiometry
TBARS	Thiobarbituric acid reactive substances
TfR1	Transferrin receptor protein 1
UV-Vis	UltraViolet-Visible Spectroscopy
y-CD	gamma-Cyclodextrin
